# Primary Neuronal Culture and Transient Transfection

**DOI:** 10.21769/BioProtoc.5169

**Published:** 2025-01-20

**Authors:** Shun-Cheng Tseng, Peng-Tzu Chen, Eric Hwang

**Affiliations:** 1 Department of Orthopedic Surgery, Changhua Christian Hospital, Changhua, Taiwan; 2 Department of Biological Science and Technology, National Yang Ming Chiao Tung University, Hsinchu, Taiwan; 3 Institute of Molecular Medicine and Bioengineering, National Yang Ming Chiao Tung University, Hsinchu, Taiwan; 4 Institute of Bioinformatics and Systems Biology, National Yang Ming Chiao Tung University, Hsinchu, Taiwan; 5 Center for Intelligent Drug Systems and Smart Bio-devices (IDS2B), National Yang Ming Chiao Tung University, Hsinchu, Taiwan

**Keywords:** Neuronal morphology, Neurite outgrowth, Neuronal isolation, Adherent neuron transient transfection, Suspension neuron transient transfection

## Abstract

Primary neuronal culture and transient transfection offer a pair of crucial tools for neuroscience research, providing a controlled environment to study the behavior, function, and interactions of neurons in vitro. These cultures can be used to investigate fundamental aspects of neuronal development and plasticity, as well as disease mechanisms. There are numerous methods of transient transfection, such as electroporation, calcium phosphate precipitation, or cationic lipid transfection. In this protocol, we used electroporation for neurons immediately before plating and cationic lipid transfection for neurons that have been cultured for a few days in vitro. In our experience, the transfection efficiency of electroporation can be as high as 30%, and cationic lipid transfection has an efficiency of 1%–2%. While cationic lipid transfection has much lower efficiency than electroporation, it does offer the advantage of a higher expression level. Therefore, these transfection methods are suitable for different stages of neurons and different expression requirements.

Key features

• Culture of primary neurons from the CNS.

• Electroporation for freshly isolated neurons in suspension.

• Cationic lipid transfection for adherent neurons.

## Graphical overview


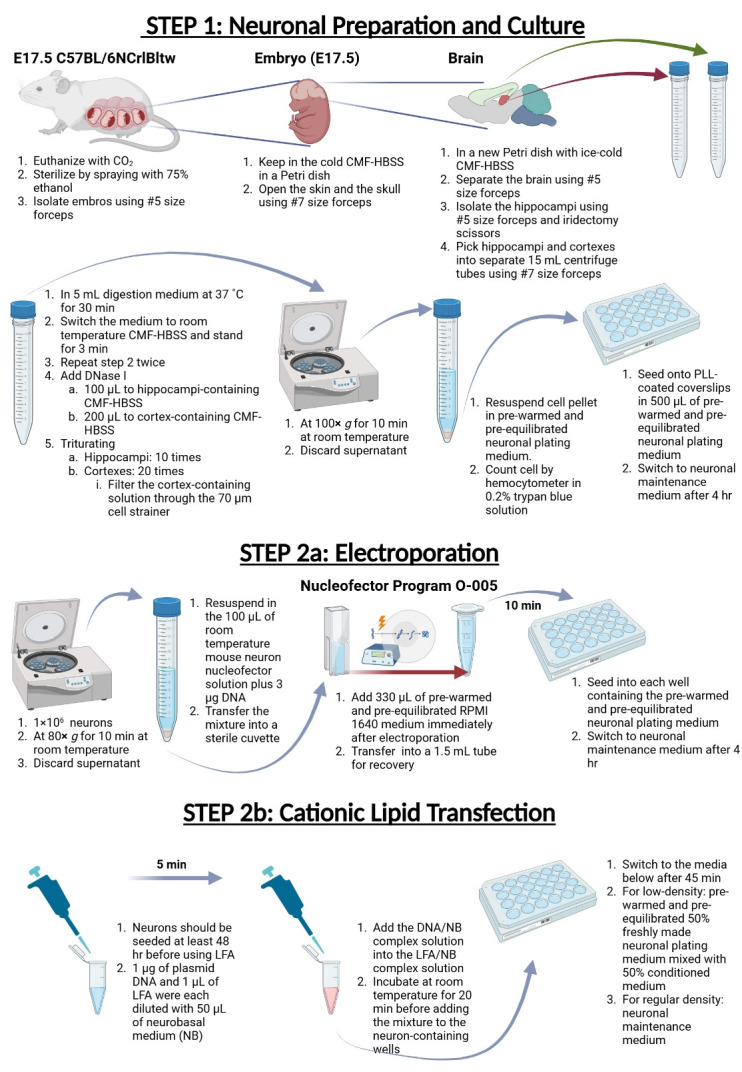


## Background

Neurons are highly specialized cells whose development and function are influenced by complex interactions of genetic and environmental factors. In vitro culture systems offer a controlled environment for studying neuroscience at the cellular level. While primary neuronal culture has its limitations (e.g., losing the complexity of the in vivo network or the stiffness of the culture surface being different from that of the nervous system), it offers a controlled environment to assess of effect of biological, chemical, or physical perturbations. To study the physiological roles of target proteins, we need to alter the expression or sequence of the target genes in neurons through transfection. Electroporation and cationic lipid transfection are two non-viral methods for introducing exogenous DNA into primary neurons. Both methods have been widely used, and their efficacy varies significantly depending on the types and developmental stages of neurons [1].

Electroporation has the advantage when the target cells are freshly isolated neurons or cells in suspension that have yet to produce cellular protrusions [1]. Electroporation can provide high transfection efficiency [2] and can be applied to a wide range of biological studies because of its versatility [3]. Electroporation is not a time-consuming procedure, allowing for efficient transfection of large numbers of cells [4], and can directly deliver exogenous DNA into cells without viral or non-viral vectors [2]. Compared to other methods of gene delivery, electroporation is generally less toxic than others [2]. It has the potential to be utilized not only for DNA transfer but also for other charged macromolecules (such as chemical molecules, antibodies, antisense oligonucleotides, RNAs, and artificial chromosomes) into neurons [5].

However, electroporation is not a perfect method for neurons at later stages of development. Adherent neurons with neurites are highly sensitive to physical stress, including the electric pulses used in electroporation [6]. In primary neuronal cultures that have been maintained in vitro for a few days, the close proximity of somata, axons, and dendrites can alter the electric field experienced by each cell compared to low-density cell suspensions. Furthermore, the presence of extracellular matrix in cultures can further influence the electroporation process [7]. To transfect neurons that have been cultured in vitro for a few days, cationic lipid transfection is a good alternative. Cationic lipid transfection achieves reliable efficiency in adherent neurons with neurites due to its ability to form unilamellar liposomes that facilitate the entry of nucleic acids into the cells. Cationic liposomes, with diameters between 100 and 500 nm, are lipid spheres with positive charges on their surface [8], which are attracted to the negative charges of both DNA and the cell membrane of neurons. This makes cationic lipid transfection less dependent on the physical properties of the cells, making it more suitable for complex cell types [9]. Unlike electroporation, cationic lipid transfection does not involve the application of electric pulses. This reduces the physical stress on neurons and improves the survival rate of the cells [2]. Additionally, cationic lipid transfection can result in higher gene expression than electroporation in our hands.

Electroporation is a powerful tool for transfection in freshly isolated neurons because of its high efficiency, while cationic lipid transfection offers a higher gene expression in adherent neurons.

## Materials and reagents

**Animals**

1. Pregnant (E17.5) C57BL/6NCrlBltw mice (BioLASCO Taiwan Co., C57BL/6)

**Reagents**

1. Fetal bovine serum (FBS) (Thermo Fisher, catalog number: 10437028)

2. B27 supplement 50× (Thermo Fisher, catalog number: 17504044)

3. Lipofectamine 2000 (LFA) (Thermo Fisher, catalog number: 11668027)

4. Minimum essential medium (MEM) (Thermo Fisher, catalog number: 11090099)

5. Neurobasal medium (NB) (Thermo Fisher, catalog number: 21103049)

6. 0.25% trypsin-EDTA (Thermo Fisher, catalog number: 25200056)

7. RPMI 1640 medium (Thermo Fisher, catalog number: 21870076)

8. Mouse Neuron Nucleofector kit (Lonza, catalog number: VPG-1001)

9. 10× Hank's balanced salt solution (HBSS) (Thermo Fisher, catalog number: 14185052)

10. 1 M N-2-hydroxyethylpiperazine-N-2-ethane sulfonic acid (HEPES) (Thermo Fisher, catalog number: 15630080)

11. Penicillin–streptomycin (10,000 U/mL) (Thermo Fisher, catalog number: 15140122)

12. D-glucose (Sigma-Aldrich, catalog number: G8769)

13. Boric acid (J.T. Baker, catalog number: 4035-01)

14. Sodium tetraborate (Sigma-Aldrich, catalog number: 31457)

15. Poly-L-lysine, MW 30,000–70,000 (Sigma-Aldrich, catalog number: P2636)

16. DNase I (Sigma-Aldrich, catalog number: DN25)

17. L-glutamine (Sigma-Aldrich, catalog number: G8540)

18. Acetone (Sigma-Aldrich, catalog number: 32201)

19. Ethanol absolute (Sigma-Aldrich, catalog number: 32221)

20. Trypan blue (Thermo Fisher, catalog number: 12250061)

**Solutions**

1. Boric acid buffer (see Recipes)

2. Poly-L-lysine stock solution (see Recipes)

3. Calcium- and magnesium-free HBSS (CMF-HBSS) (see Recipes)

4. DNase I solution (see Recipes)

5. Digestion medium (see Recipes)

6. L-glutamine solution (see Recipes)

7. Neuronal plating medium (see Recipes)

8. Neuronal maintenance medium (see Recipes)

**Recipes**

**1. Boric acid buffer**

**Table:** 

Reagent	Final concentration	Quantity or Volume
Boric acid	50 mM	1.24 g
Sodium tetraborate	12 mM	1.90 g

Adjust to pH 8.5 with 1 M NaOH, add sterile ddH_2_O to 400 mL, and store at 4 °C.

**2. Poly-L-lysine stock solution**

**Table:** 

Reagent	Final concentration	Quantity or Volume
Poly-L-lysine	1 mg/mL	50 mg
Boric acid buffer		50 mL

Use the top bottle filter to sterilize and store at 4 °C.

The working solution is made by diluting the stock solution with boric acid buffer to 100 μg/mL.

**3. Calcium- and magnesium-free HBSS (CMF-HBSS)**

**Table:** 

Reagent	Final concentration	Quantity or Volume
1 M HEPES	10 mM	5 mL
Penicillin–streptomycin (10,000 U/mL)	100 U/mL	5 mL
10× HBSS	1×	50 mL

Add sterile ddH_2_O to 500 mL and store at 4 °C.

**4. DNase I solution**

**Table:** 

Reagent	Final concentration	Quantity or Volume
DNase I	10 mg/mL	10 mg
ddH_2_O		1 mL

Aliquot into 1.5 mL microcentrifuge tubes and store at -20 °C.

**5. Digestion medium**

**Table:** 

Reagent	Final concentration	Quantity or Volume
1 M HEPES	10 mM	1 mL
0.25% trypsin-EDTA		100 mL

Aliquot into 15 mL centrifuge tubes and store at -20 °C.

**6. L-glutamine solution**

**Table:** 

Reagent	Final concentration	Quantity or Volume
L-glutamine	200 mM	1.46 g

Add ddH_2_O to 50 mL. Warm up in a 37 °C water bath for 30 min, vortex the tube every 5 min to ensure L-glutamine is dissolved, aliquot into 1.5 mL microcentrifuge tubes, and store at -20 °C.

**7. Neuronal plating medium**

**Table:** 

Reagent	Final concentration	Quantity or Volume
D-glucose	0.6%	200 μL
FBS	5%	750 μL
L-glutamine solution	2 mM	150 μL

Add MEM so the final volume is 15 mL.

**8. Neuronal maintenance medium**

**Table:** 

Reagent	Final concentration	Quantity or Volume
L-glutamine solution (200 mM)	0.5 mM	125 μL
B27 supplement 50×	1×	1 mL

Add neurobasal medium so the final volume is 50 mL. B27 is light-sensitive.

**Laboratory supplies**

1. Untreated polystyrene 24-well plate (Corning, catalog number: 351147)

2. 12 mm round glass coverslips (Marienfeld, catalog number: 0111520)

3. 70 μm cell strainer (Corning, catalog number: 352350)

4. 0.22 μm 500 mL PES top bottle filter (Corning, catalog number: COR431118)

5. 0.22 μm PES syringe filter (Sartorius, catalog number: 16532-k)

6. 50 mL syringe without needle (TERUMO, catalog number: 2TEM-SS50LZ/12)

7. 10 cm cell culture dish (Corning, catalog number: COR-430167)

8. 9 cm Petri dish (UR Brand, catalog number: UR-PD15R-1BOX)

9. 50 mL centrifuge tube (Corning, catalog number: 430829)

10. 15 mL centrifuge tube (Corning, catalog number: 430791)

11. 1.5 mL microcentrifuge tube (SSI Bio, catalog number: 1SSI-1260-00)

## Equipment

1. Dissection microscope (Nikon, model: SMZ745)

2. Dissection tools:

a. Dumont #5 forceps (Roboz Surgical Instrument, model: RS-5045)

b. Dumont #7 forceps (Roboz Surgical Instrument, model: RS-5047)

c. Iris scissors (Dimeda Surgical Instruments, model: 08.340.11)

d. Iridectomy scissors (Dimeda Surgical Instrument, model: 09.140.08)

3. Laminar flow cabinet (Tsaohsin Enterprise, model: TH-420)

4. Biosafety cabinet (Esco, model: AC2-462)

5. Tabletop centrifuge (Kendro laboratory product, model: Legend RT)

6. Hemocytometer (Marienfeld, model: AP73811-00710)

7. Amaxa Nucleofector^TM^ system (Lonza, model: Amaxa Nucleofector II)

8. CO_2_ incubator (NuAire, model: NU-8500)

9. Water bath (Firstek, model: B206-T1)

## Software and datasets

1. BioRender (https://biorender.com). The following figures were created using BioRender: The graphical overview is created in BioRender by Tseng, L. (2024), https://BioRender.com/w09e536.

## Procedure

**A. Preparation of coverslips or 10 cm cell culture dish**

1. Wash 12 mm coverslips once with acetone and twice with absolute ethanol on a shaker (90 rpm) for 15 min at room temperature.

2. After the second wash with ethanol, transfer coverslips into the laminar flow cabinet and then wash with sterile ddH_2_O thrice.

3. Transfer coverslips into each well of a 24-well plate. Remove ddH_2_O using aspiration. For 24-well plates, apply 200 μL of 100 μg/mL poly-L-lysine (PLL) solution to each well and incubate overnight at room temperature for coverslip coating. For 10 cm cell culture dishes, add 7 mL of 100 μg/mL PLL solution. Avoid UV light exposure after PLL was applied.

4. After PLL coating, wash coverslips with ddH_2_O twice and air dry.

5. PLL-coated coverslips or dishes can be kept in the refrigerator for up to one week.

**B. Neuron preparation and culture for primary hippocampal and cortical neurons**

1. Euthanize pregnant E17.5 C57BL/6NCrlBltw dams with CO_2_.

2. Sterilize the abdominal fur by spraying with 75% ethanol.

3. Cut the fur, exposing the skin and muscle layer.

4. Isolate the uterus with embryos from the abdominal cavity using the Dumont #5 forceps.

5. Take out embryos from the uterus using Dumont #5 forceps and remove the placentas. Keep in ice-cold CMF-HBSS in a Petri dish before the brain dissection.

6. Hold the E17.5 embryos by the neck with Dumont #5 forceps and use the tip of the Dumont #7 forceps to gently cut open the skin and the skull. Carefully cut open the skull from the nose to the back of the head and squeeze out the brain by pinching the sides of the skull.

7. Place the embryonic brains in a new Petri dish filled with ice-cold CMF-HBSS.

8. Operating under a stereomicroscope, separate brain cortexes from each brain and remove the meninges using a pair of Dumont #5 forceps.

9. Dissect hippocampi from both cortical hemispheres and remove the fimbriae on the concave side using the Dumont #5 forceps and the iridectomy scissors.

10. Transfer hippocampi and cortexes using Dumont #7 forceps into two different 15 mL centrifuge tubes containing 5 mL of digestion medium and incubate at 37 °C for 30 min.

11. Replace the digestion medium with room-temperature CMF-HBSS and let it stand for 3 min. Repeat the step twice to allow the residual digestion medium to diffuse from the tissue.

12. Apply 100 μL of DNase I solution to the hippocampi-containing CMF-HBSS and 200 μL of DNase I solution to the cortex-containing CMF-HBSS.

13. Then, triturate hippocampi 10 times with a 10 mL serological pipette and 10 times with a 10 mL serological pipette tipped with a 1,000 μL tip until the tissue dissociates. Triturate cortexes 20 times with a 10 mL serological pipette and 20 times with a 10 mL serological pipette tipped with a 1,000 μL tip until the tissue dissociates. Then, filter the tissue through the 70 μm cell strainer to remove tissue debris.

14. Centrifuge dissociated neurons at 100× *g* for 10 min at room temperature and discard the supernatant.

15. Dissociate the cell pellet by flicking the bottom of the microcentrifuge tube a few times and resuspending in the 37 °C prewarmed and pre-equilibrated neuronal plating medium.

16. Determine cell number using a hemocytometer in the presence of 0.2% trypan blue solution.

17. Seed dissociated neurons onto poly-L-lysine-coated coverslips (2.5 × 10^3^ cells/cm^2^ for low-density cultures and 3 × 10^4^ cells/cm^2^ for regular-density cultures) in 500 μL of prewarmed and pre-equilibrated neuronal plating medium.

18. After 4 hours, replace the neuronal plating medium with the prewarmed and pre-equilibrated 50% freshly made neuronal plating medium mixed with 50% conditioned medium (for low-density cultures) or neuronal maintenance medium (for regular-density cultures). To prepare the conditioned medium, seed 2.2 × 10^6^ cortical neurons in a poly-L-lysine-coated 10 cm cell culture dish and culture for 7 days. The cell culture medium is the conditioned medium; it can be stored at -20 °C for several months.

**C. Electroporation**

1. Centrifuge 1 × 10^6^ dissociated neurons at 80× *g* for 10 min at room temperature and remove the supernatant.

2. Carefully resuspend the neuron pellet in 100 μL of room-temperature mouse neuron nucleofector solution plus 3 μg of DNA. Then, transfer the mixture into a sterile cuvette.

3. Perform electroporation with the Nucleofector Program O-005 for mouse neuron nucleofector.

4. Add 330 μL of prewarmed and pre-equilibrated RPMI 1640 medium to a cuvette immediately after electroporation and transfer gently (without pipetting up and down) into a 1.5 mL tube for recovery (incubating for 10 min in a 37 °C CO_2_ incubator).

5. After the recovery step, seed neurons into each well containing the prewarmed and pre-equilibrated neuronal plating medium using the aforementioned density.

6. After 4 h, replace the neuronal plating medium with the fresh neuronal maintenance medium.

7. The protein expressed from the plasmid can usually be detected in 24 h.

**D. Cationic lipid transfection**

1. Transfect neurons in each well at least 48 h after seeding using Lipofectamine 2000 (LFA). In our experience, transfecting neurons 48 h after seeding causes severe cell death.

2. For one reaction, dilute 1 μg of plasmid DNA and 1 μL of LFA with 50 μL each of neurobasal medium (NB) to make the DNA/NB complex solution and the LFA/NB complex solution. This plasmid DNA-to-LFA ratio must be empirically determined for optimal transfection efficiency.

3. After 5 min, pipette the DNA/NB complex solution into the LFA/NB solution. Mix the mixture by flicking the bottom of the tube and leave to stand at room temperature for 20 min before adding it to each neuron-containing well.

4. Forty-five minutes after transfection, replace the medium with prewarmed and pre-equilibrated 50% freshly made neuronal plating medium mixed with 50% conditioned medium (for low-density cultures) or neuronal maintenance medium (for regular-density cultures).

5. The protein expressed from the plasmid can usually be detected in 24 h.

## Data analysis

**Figure 1.**
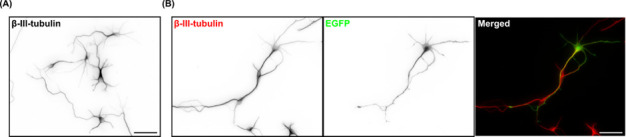
Morphology and expression of EGFP in primary cortical neurons. (A) Representative image of 3 days in vitro (DIV) primary cortical neurons immunofluorescence stained with the neuron-specific β-III-tubulin antibody. (B) Representative image of 3 DIV primary cortical neurons immunofluorescence stained with β-III-tubulin (red in the merged image) and EGFP (green) antibodies. Neurons were transfected with a cytosolic EGFP-expressing plasmid on 2 DIV using Lipofectamine 2000. Scale bars represent 50 μm.

Neurons can be fixed at specific days in vitro (DIV) for subsequent image analyses. For transfection efficiency quantification, neurons were transiently transfected with a plasmid expressing the cytosolic EGFP and immunofluorescence stained with antibodies against neuron-specific β-III-tubulin and the transfection indicator EGFP (Figure 1). At least 2.4 × 10^5^ neurons (or the number of neurons on four coverslips) were counted to calculate the transfection efficiency. When the mean fluorescence intensity of the EGFP signal in the soma of a particular neuron was higher than three times the standard deviation of the mean fluorescence intensity of the background, that neuron was considered transfected. For morphological analyses, we typically use the SNT plugin for ImageJ or Fiji [10].

## Validation of protocol

This protocol or parts of it have been used and validated in the following research article(s):

• Chen et al. [11]. The non-mitotic role of HMMR in regulating the localization of TPX2 and the dynamics of microtubules in neurons. *eLife* 13. https://doi.org/10.7554/eLife.94547

## General notes and troubleshooting

**General notes**

1. For quality control, the number of harvested cells should be consistent, with approximately 1 × 10^7^ cortical neurons from three embryonic mouse brains.

2. For optimal trypsin digestion, the number of cortices per tube should be between 3 and 4 to prevent incomplete trypsin digestion.

3. Generally, healthy neurons should adhere to the plate within 2 h after seeding, and structures similar to lamellipodia and filopodia should appear approximately 4 h later.

4. After electroporation, the survival rate of neurons is approximately 50%. As a result, one needs to increase the seeding density to compensate for this.

5. The DNase I solution should be prepared on the same day of the experiment and stored in a 4 °C refrigerator before the experiment.

6. The entire dissection procedure should be performed in ice-cold CMF-HBSS.

7. Neuronal plating medium, neuronal maintenance medium, and RPMI 1640 medium should be placed in a CO_2_ incubator for at least 15 min for pH pre-equilibrium.

8. Adding the conditioned medium promotes neurite outgrowth and axonal formation. Those who are studying the morphogenetic process of neurons should be aware of this.

9. To reduce the possibility of contamination between tissues, it is recommended that separate surgical instruments be used for different dissection steps.

**Troubleshooting**

Problem 1: During dissection, the anatomical structure of the hippocampus is unclear, making it difficult to distinguish and separate it from the surrounding tissues.

Possible cause: Insufficient gestation period.

Solution: Confirm the calculation method of the mouse gestation period with the supplier and record data such as the number and size of embryos.

Problem 2: Dissociated cells do not completely precipitate after the centrifugation step.

Possible cause: Tissue dissociation is incomplete.

Solution: Invert the centrifuge tube to facilitate the dissociation of cells and centrifuge again.

Problem 3: Neurons are not completely dissociated after seeding.

Possible cause: Cells were not completely dissociated before replacing the digestion medium with neuronal plating medium after centrifugation.

Solution: Try tapping the centrifuge tube to disperse the cell pellet after removing the digestion medium.

Problem 4: The somata are clustered, and the neurites are bundled together.

Possible cause: Problem with PLL coating or the glass coverslip.

Solution: The quality of the glass is crucial; use borosilicate glass for the coverslip. Wash the coverslips thoroughly and do not store washed coverslips for too long before use. Make sure to use the PLL with proper molecular weight (30,000–70,000) and use the PLL-coated coverslip within one week.
